# An Account of a Recent Epidemic of Scarlatina *in Mercer and Venango Counties, Pa.*

**Published:** 1839-11-23

**Authors:** E. W. Glizen

**Affiliations:** Mercer, Pa.


### AN ACCOUNT OF A RECENT EPIDEMIC OF SCARLATINA *in Mercer and Venango Counties, Pa.* By E. W. Glizen, M. D.

To the Editors of the Medical Examiner.

Gentlemen,—My object in the present communication is to offer you an account of an epidemic of scarlatina, which has been for some time prevailing in the eastern part of Mercer, and western part of Venango counties, in Pennsylvania.

As usual, the three varieties of the disease were presented, in different individuals, in many of the same families. So, likewise, in some the scarlet eruption was present, and in some it was wanting, though there was a greater proportion of general eruptive cases in this epidemic than is usual in epidemics of the disease in this region. The malignant cases which I saw were rendered so, either by an attack of vomiting and purging, or by the use of too active emetics and cathartics. Some cases, which were ushered in by vomiting and purging, terminated fatally in twenty-four and forty-eight hours. Others put on all the characters of malignant scarlatina,—great prostration of strength; more or less coma; small and frequent pulse; imperfect reaction; imperfect appearance, and disappearance of the eruption; coldness of the extremities; extensive inflammation and ulceration of the tonsils, extending up into the posterior nares, and discharging a thin coryza, which excoriated the parts; great precordial distress; countenance sunk, and lips purple. Some of these cases lasted six and seven days.

I witnessed several of these cases which terminated fatally, which had been in progress four and five days when I was called into the neighbourhood, which is fifteen miles east of the borough of Mercer, where I reside. The plan of treatment which I fixed upon, and which proved successful, in between forty and fifty cases, was mainly as follows:—In the malignant cases, attacked as above described, every effort was made to check the vomiting and purging speedily, by sinapisms to the stomach, feet, and legs, aqua ammonia, and warm brandy sling, camphor, and opium. As soon as this could be checked, and reaction was brought about, I commenced with small doses of calomel and ipecac., being careful not to produce nausea; at the same time I gave a tea-spoonful of a solution of common salt, and an infusion of cayenne pepper, prepared by putting a teaspoonful of cayenne and two teaspoonsful of salt into a teacup full of boiling water, together with twelve drops of aqua ammonia, every two hours. For a drink, I ordered cold water to be poured upon slippery elm bark, in which was dissolved a small quantity of nitre. This course I pursued till the patient complained of a slight soreness of the teeth; the calomel and ipecac. were then discontinued, and small, but daily doses of cold-pressed castor oil, were ordered to be continued till the stools, which were now very dark, should become natural. As a febrifuge, after the reaction was fully established, the surface was, as often as necessary to moderate the intensity of the heat, sponged over with cold water. Where any local determination appeared, as was often the case in the region of the stomach, the skin was slightly scarified, and the tartar emetic ointment applied.

The same ointment was applied externally in the same way over the tonsils. These pustules, after twelve hours, were dressed with cabbage leaves. The only thing to guard against in the application of the tartar emetic ointment over the tonsils, is the production of too deep sores, and consequent scars.

In those cases in which the reaction was vigorous, the use of the cayenne was evidently hurtful. My practice in these cases was to give of calomel and ipecac. each three grains, at bedtime, to be followed in the morning with castor oil,—and through the day, if the pulse was high, and the heat of the surface intense, I gave divided doses of ipecacuanha at intervals of one or two hours, till the patient vomited. The calomel and ipecac. I continued till the gums were touched, or the fever abated. The ipecac. and castor oil were used daily till all evidence of fever disappeared.

The tartar emetic ointment, or the Croton oil to pustulate, was applied over the tonsils, in every variety, and always with immediate relief to the throat. It appeared to me that the cayenne and salt were useful only in the malignant cases, and those cases in which it evidently kept up a perspiration; in all other cases, especially where the action was vigorous, it evidently increased the pain and inflammation of the throat, and indeed aggravated every symptom.

In fine, the whole object was to preserve a medium of action, and correct the secretions. The internal use of the tartar emetic, to any extent, was most injurious. The spongings with cold water were frequently attended with the happiest effects in allaying that restlessness produced by the heat and eruption upon the surface.

*Mercer, Pa.*, September 24th, 1839.

